# 1221. Non-severe, non-immune mediated intolerances: simple targets for direct non-invasive delabeling opportunities of beta-lactam antibiotic allergy labels (BAAL)

**DOI:** 10.1093/ofid/ofad500.1061

**Published:** 2023-11-27

**Authors:** Radhika S Polisetty, Michael Dickens, Hoang Tran, Nour Khankan, Erin Weslander, Christie M Bertram, William J Moore, Sheila K Wang

**Affiliations:** Midwestern University College of Pharmacy/ Northwestern Medicine Central DuPage Hospital, Winfield, Illinois; Northwestern Memorial Hospital, IL, Illinois; Midwestern University College of Pharmacy, Downers Grove Campus, Downers Grove, Illinois; Midwestern University, Downers Grove, Illinois; Northwestern Memorial Hospital, IL, Illinois; Northwestern Memorial Hospital/Rosalind Franklin University of Medicine and Science, Chicago, Illinois; Northwestern Medicine, Chicago, Illinois; Midwestern University College of Pharmacy/Northwestern Memorial Hosptial, Downers Grove, Illinois

## Abstract

**Background:**

Majority of patients with a BAALs can tolerate a β-lactam without concerns for a severe IgE-mediated reaction. Many BAALs are confirmed to be intolerances or adverse events versus a true allergy. False BAALs can result in the use of alternative non-BLs, posing risk for collateral health consequences. Current strategies to delabel a false BAAL can be restricted, time consuming and invasive, requiring skin and drug provocation testing. An alternative approach involves direct delabeling of reported BL allergies in patients with non-severe, non-immune mediated intolerances. This method of assessment relies on the review of the electronic medical record (EMR) versus invasive assessments. The aim of this study was to identify the prevalence of reported non-severe, non-immune mediated intolerances across a system of hospitals for prospective non-invasive delabeling opportunities.

**Methods:**

Eligible patients seen within the Northwestern Medicine Healthcare System (NMHS) between 8/1/20 and 8/1/22 were included. Eligible patients: 18 years and older, admitted to a NMHS hospital with > 1 overnight stay, and a BAAL in the allergy profile of the EMR. Examples of non-severe, non-immune mediated intolerances were GI upset, rash w/o pruritus, headache, and pharmacologically predictable reactions. Demographic and allergy data reported in the allergy profile of the EMR was retrospectively obtained through from the Epic Enterprise Data Warehouse.

**Results:**

Over the study period, 25,768 unique patients were eligible for inclusion. During this time, the NMHS admitted 194,670 patients, suggesting a BAAL prevalence rate of 13.2% system-wide. Included patients were mostly White (81%), female (65%) with a median age of 64 (18-109). Of the 25,768 BAALs, 16,561 (64%) were reported as immune mediated reactions, 2,534 (9.8%) as non-severe, non-immune mediated intolerances, 3,405 (13.2%) as indeterminant, and 3,268 (12.7%) had no reaction type reported in the allergy profile of the EMR.

**Conclusion:**

Our prevalence of BAAL across NMHS hospitals was 13.2%. At least 2,534 BAALs reported as a non-severe, non-immune mediated intolerance could be directly delabeled using a non-invasive approach, possibly offering a simpler and broader approach compared to current (invasive) strategies.

**Disclosures:**

**All Authors**: No reported disclosures

